# The magnitude of undiagnosed diabetes and Hypertension among adult psychiatric patients receiving antipsychotic treatment

**DOI:** 10.1186/s13098-020-00588-2

**Published:** 2020-09-07

**Authors:** Agete Tadewos Hirigo, Tesfaye Teshome

**Affiliations:** 1 College of Medicine and Health Science, Faculty of Medicine, School of Medical Laboratory Sciences, Hawassa University, P.O. Box 1560, Hawassa, Ethiopia; 2 College of Medicine and Health Science, Faculty of Medicine, Physiology unit, Hawassa University, Hawassa, Ethiopia

**Keywords:** Severe mental illness, Hypertension, Diabetes

## Abstract

**Background:**

Patients with severe mental illness (SMI) are at increased risk of developing non-communicable diseases that could cause significantly lower life expectancy when compared to the general population. This study aimed to assess the magnitude and predictors of undiagnosed type-2 diabetes and hypertension among adult patients with SMI on antipsychotic treatments.

**Methods:**

A hospital-based cross-sectional study was conducted on 237 psychiatric patients from January to June 2019 at Hawassa University Comprehensive Specialized Hospital, Hawassa, Southern Ethiopia. All relevant information was collected using a structured interviewer-administered questionnaire with a systematic random sampling technique. A total of 4–5 mL of overnight fasting venous blood was collected from each patient. Serum lipid profiles and fasting blood sugar (FBS) were measured using the A25™ BioSystem Random Access chemistry analyzer. To identify predictors of hyperglycemia and raised blood pressure, multiple linear regression analysis was done using SPSS version 23. Statistical significance was set at p value < 5%.

**Results:**

From 247 patients with SMI approached, 237 (58.2% male and 41.8% females) were take part in the study giving a response rate of 95.9%. The overall 31.2% (95%CI: 24.1–37.6) and 27.8% (95%CI: 23.2–33.4) of patients had hyperglycemia and raised BP. The magnitude of prediabetes and type-2 diabetes was 24.9% (95%CI:19.4–30.4), and 6.3% (95% CI: 3.4–10.1), respectively. While the magnitude of prehypertension and hypertension was 23.2% (95%CI: 17.3–29.5) and 4.6% (95%CI: 2.1–8.0), respectively. In multiple linear regression analyses: age, HDL-cholesterol, physical activity and Triglyceride/HDL-cholesterol ratio were positively correlated with FBS. While, HDL-cholesterol, waist circumference, physical activity, total cholesterol/HDL-c ratio, and body mass index were positively correlated with systolic and diastolic blood pressures.

**Conclusion:**

The findings indicate a need to assess blood glucose and blood pressure at baseline before the commencement of any antipsychotic therapy and during therapeutic follow up to manage any increasing trends. Moreover, close monitoring of patients with severe mental illness on antipsychotic therapy is exclusively recommended.

## Background

Patients with severe mental illnesses [SMI] have a high risk of developing metabolic disturbances that include obesity, type-2 diabetes, dyslipidemia, hypertension (HTN) and metabolic syndrome [1]. Besides, patients with SMI have two to three times higher chance of mortality rate and significantly low life expectancy when compared to the general population [2, 3]. This is due to the presence of existing comorbidities with SMI like diabetes mellitus, HTN, obesity and lack of attention in the mental health clinics especially in developing and low-income countries [4, 5]. Patients with SMI could be affected highly with type-2 diabetes and hypertension [6, 7].

HTN can affect 35%-61% of patients with bipolar disorder and 19%-58% of schizophrenic patients [8]. Besides, the prevalence of type-2 diabetes ranges from 10–15% in schizophrenic patients and 8–17% among patients with bipolar disorder [8]. The study report from London UK indicated 16% of the overall estimated prevalence of Type 2 diabetes in people with severe mental problems [9]. While other study conducted in South Africa indicated about 32% and 8% prevalence of HTN and type-2 diabetes among long-term psychiatric patients, respectively [10].

Different studies that conducted in Ethiopia showed the prevalence of type-2 diabetes in the general population ranging from 1.9% -12.2%, like 1.9% [11], 6.5% [12] and 12.2% [13] in South region of Ethiopia, 3.3% [14], 6.8% [15] and 10.2% [16] in Amhara region, and 6.5% in Addis Ababa [17]. While, the prevalence of HTN in the general population was 10.5% in Hawassa, South Ethiopia [13], 19.1% in Addis Ababa, Ethiopia [17], 24.43% in Dire Dawa, East-Ethiopia [18], 16% in Tigray, North-Ethiopia [19] and 12.5% in Debre Markos, Northwest Ethiopia [20].

Different studies highlighted an elevated prevalence of type-2 diabetes [13, 16] and HTN [13, 17–19] among patients with SMI receiving antipsychotic therapy. However, only one study revealed a 7.3% prevalence of undiagnosed type-2 diabetes among psychiatric patients in Ethiopia [21] and data regarding undiagnosed type-2 diabetes and HTN among the depicted patients in Ethiopia are very scarce. Therefore, this study aimed to assess the magnitude and its predictors of undiagnosed type-2 diabetes and HTN among adults with SMI receiving antipsychotic therapy.

## Methods

### Study setting, design, and population

This study was conducted in Hawassa University comprehensive specialized Hospital, Southern Nations Nationalities, and Peoples Region (SNNPR) from January to June 2019. Hawassa is the capital city of the SNNPR region and located 275 km distant from Addis Ababa, the capital city of Ethiopia. This institution based cross-sectional study was conducted among psychiatric patients who had a minimum of one-year regular follow up in the psychiatric department. All patients with SMI, aged greater than or equal to 18 years and who had received the current antipsychotic medication at least for 12 months were eligible for the study. In addition, stabilizing treatment was given for patients before consenting to the study. However, patients with previously diagnosed and known type-2diabetes and HTN were not included in the study based on the objective of this study.

### Sample size and sampling technique

The sample size assumption was based on a 16% proportion of type-2 diabetes among psychiatric patients using a single population proportion formula at 95% confidence interval (CI) and marginal error of 5% [9]. In addition, a 20% non-response rate was considered and the final sample size calculated to be 247. A systematic random sampling technique was applied to select the study participants from psychiatric patients in the psychiatric outpatient department.

### Definition of terms

#### Severe mental illness

Its length of disease and the disability it produces often defines it. The illnesses include disorders that produce psychotic symptoms, such as schizophrenia, schizoaffective disorder, and severe forms of other disorders, such as major depression and bipolar disorders. In addition, the illnesses that produce distortions of perception, delusions, hallucinations, and unusual behaviors are sometimes called thought disorders [22].

According to the American Diabetes Association (ADA) criteria, prediabetes (impaired fasting glucose) is defined as fasting blood sugar (FBS) 100–125 mg/dl; whereas type 2-diabetes is defined as the presence of FBS ≥ 126 mg/dl [23]. Undiagnosed type-2 diabetes is defined as patients having formerly undiagnosed or unknowingly elevated FBS level that desired the minimum concentration (≥ 126 mg/dl) to attain diabetes definition [23].

Pre-HTN is defined as systolic blood pressure (SBP) 120–139 mmHg and a diastolic blood pressure (DBP) 80–89 mmHg in the absence of any result influencing factors [24]; whereas HTN is defined as SBP ≥ 140/DBP ≥ 90 mmHg [24]. In addition, undiagnosed HTN is defined as patients having unknowingly elevated blood pressure that desired the minimum level (BP ≥ 140/90 mmHg) to attain the HTN definition [24].

### Data collection and assessments

Socio-demographic data like age, sex, education, marital status, ethnicity, occupation and religion; behavioral data such as khat chewing, smoking, and alcohol taking and clinical data like FBS, BP, type of mental disorder, duration of disease and type of treatments were collected by the interviewer-administered structured questionnaires. Before measuring BP, each patient was asked regarding alcohol/caffeine intake, smoking, stressful condition, and bathing for 30 min before taking a measurement. Then psychiatric nurses who were working in the psychiatric department measured each participant’s blood pressure from the left arm in sitting position. In addition, two BP measurements was taken from each patient within a 3-min difference using a digital electronic sphygmomanometer (Omron, Healthcare, Japan) and then the average was taken to address individuals' BP status. Moreover, weight and height measurements were taken from the  individuals based on the WHO stepwise guideline [25], using an Adult's digital electronic scale that has both weight and height scales. Body mass index (BMI) was calculated as (weight/height^2^) and classified as BMI < 18.5 kg/m^2^ for underweight, 18.5–24.9Kg/m^2^ for normal weight, 25–29.9 kg/m^2^ for overweight and ≥ 30 kg/m^2^ for obesity [26]. Furthermore, the WHO stepwise technique [27] was applied to measure WC of the patients with non-stretching tape.

Overnight fasting blood was collected from each participant for determining serum lipid profiles (total cholesterol [TC], HDL-cholesterol [HDL-c], and triglycerides [TGs]) and FBS using A25™ BioSystem Random Access chemistry analyzer. All chemicals for serum biochemical analysis were from Linear manufacturers (Linear chemicals, Montgat, Spain).

### Statistical analysis

Data of each questionnaire was checked, entered into and analyzed using Statistical Package for Social Sciences (SPSS) Version 23. Descriptive statistics such as frequency and percentages were applied to summarize categorical variables. Besides normally distributed continuous variables were tabulated via means and standard deviation, while median and interquartile range (IQR) were applied for data with skewed distribution. Pearson's correlation coefficient was used to find out the relationship of FBS, SBP, and DBP with different independent variables in all groups. Linear regression models also were analyzed to find out the independent factors that affecting FBS, SBP and DBP, and to determine significant predictors. It was accepted statistically significant when a p-value < 0.05 at 95% CI.

### Data quality

Quality of data collection tool was checked by pretesting of 10% questionnaires before conducting actual data collection. All essential restructuring of the questionnaire was done based on the pretest response. Trained nurses who were working in psychiatric departments participated in data collection; while laboratory technologists manage blood samples collection as well as laboratory diagnoses. During the initial day of contact, patients were advised to come with overnight fasting for the subsequent appointment, and only patients come with overnight fasting gave blood samples. Commercially prepared quality control samples that contained the target values were performed daily before running the patients’ samples to ensure the proper functioning of chemistry analyzer, laboratory chemicals and technical performance with the strict adherence of standard operating procedures (SOPs).

## Results

### Socio-demographic and behavioral characteristics of the study population

Out of the total 247 patients with severe mental illness approached, 10 of them were excluded from the study (4 patients were known type-2 diabetic, 4 patients were known hypertensives and 2 patients were refused to take part in the study). The overall 237 [138 (58.2%) males and 99 (41.8%) females] have participated in the study with a mean age of 31.7 ± 10.9 years old. The median duration since the diagnosis of SMI was 3 and raging from 1 to 16 years, while the mean duration since the starting of antipsychotic treatment was 4.8 (± 2.6) years. In addition, 129 (54.4%) of participants were protestant in religion, 29.1% were jobless and 22.8% were students. Of the total patients, about 15 (7.2%) were smokers, 21 (8.9%) were khat chewers, and 14 (5.9%) were drinking alcohol. Majority of the patients, 203 (85.7%) had inactive lifestyle, 53 (22.3%) were overweight to obese (BMI ≥ 25 kg/m^2^) and 26(11%) were underweight (BMI < 18.5 kg/m^2^) (Table 1). Of the total 125 males: 9.4%, 11.6%, and 17.4% were drinking alcohol, smoking cigarette, and had sedentary lifestyles, while of 89 female participants: 1%, 1%, and 4.2% were drinking alcohol, smoking cigarette and had sedentary lifestyle. Furthermore, 22.8%, 19.4%, and 34.2% of participants were using chlorpromazine, risperidone, and combined type of antipsychotic agents.

**Table 1:** Socio-demographic, behavioral and biochemicals characteristics of the study subjects

Variables	Category	n (%)	Variables	Category	n (%)
Sex	Female	99 (41.8)	Marital status, n (%)	Single	140 (59.1)
	Male	138 (58.2)		Married	94 (39.7)
Age, years	mean (± SD)	31.7 (10.9)		Divorced/widowed	3 (1.3)
Age, years	≤ 20	42 (17.7)	Education, n (%)	Unable to read and write	11 (4.6)
	21–30	95 (40.1)		Primary	91 (38.4)
	31–40	58 (24.5)		Secondary	54 (22.8)
	41–50	30 (12.7)		Certificate and above	81 (34.2)
	≥ 51	12 (5.1)	Smoking, n (%)	No	220 (92.8)
Disease duration since its diagnosis in years	Median (IQR)	3 (2.0–5.0)		Yes	17 (7.2)
	≤ 3	84 (35.4)	Alcohol, n (%)	No	223 (94.1)
	4–6	122 (51.5)		Yes	14 (5.9)
	≥ 7	31 (13.1)	Khat chewing, n (%)	No	216 (91.1)
Religion, n (%)	Orthodox	77 (32.5)		Yes	21 (8.9)
	Protestant	129 (54.4)	TC, mg/dl	mean (± SD)	168.2 (47.2)
	Muslim	25 (10.5)	TG, mg/dl	Median (IQR)	111 (83–153)
	Others	6 (2.5)	FBS, mg/dl	mean (± SD)	96.9 (26.7)
Ethnicity, n (%)	Sidama	67 (28.3)	WC, cm	mean (± SD)	81.7 (10.5)
	Wolayita	29 (12.2)	SBP, mmHg	mean (± SD)	119.7 (9.3)
	Amhara	49 (20.7)	DBP, mmHg	mean (± SD)	78.6 (6.7)
	Oromo	41 (17.3)	HDL-c, mg/dl	mean (± SD)	50 (17.7)
	Others	51 (21.5)	Non-HDL-TC	mean (± SD)	117 (47.2)
Occupation	Employed	54 (22.8)	TC/HDL-c ratio	mean (± SD)	3.8 (1.8)
	Merchants	16 (6.8)	TG/HDL-c ratio	Median (IQR)	2.2 (1.5–3.7)
	Farmers	16 (6.8)	BMI, Kg/m^2^	mean (± SD)	22.9 (4.7)
	Students	54 (22.8)	BMI, Kg/m^2^	< 18.5	26 (11)
	housewife	28 (11.8)		18.5–24.9	158 (66.7)
	Jobless	69 (29.1)		25–29.9	38 (16)
Exercise (moderate-intensive), n (%)	No	203 (85.7)		≥ 30	15 (6.3)
	Yes	34 (14.3)			

BMI, body mass index; DBP, diastolic blood pressure; FBS, fasting blood sugar; TC, total cholesterol; TGs, triglycerides; HDL-c, HDL-cholesterol; WC, waist circumference; SBP, systolic blood pressure; [moderate exercise, like brisk walk, hiking on a nature trail, performing chores around the house]; [intensive exercise, causes sweat profusely and makes it difficult to carry on a conversation of any length, like some examples would be running around a track, heavy manual labor, or heavy weightlifting]

### The magnitude of prehypertension, hypertension, prediabetes, and type-2 diabetes

Overall 74 (31.2%, 95%CI: 24.1–37.6) and 66 (27.8%, 95%CI: 23.2–33.4) of the study patients had hyperglycemia (FBS > 100 mg/dl) and raised BP (≥ 120/80 mmHg), respectively. In addition, 55 (23.2%; 95% CI: 17.3–29.5) participants had prehypertension and 11 (4.6%; 95%CI: 2.1–8.0) had stage-I hypertension. While, 59 (24.9%; 95%CI: 19.4–30.4) and 15 (6.3%; 95% CI: 3.4–10.1) participants had prediabetes and type 2 diabetes, respectively.

The proportion of type-2 diabetes, prediabetes, and prehypertension were higher in males, whereas the rate of hypertension was higher in females. The proportion of prediabetes, type-2 diabetes, prehypertension, and hypertension was higher among physically inactive patients when compared to those who had the experience of performing light to moderate physical exercise (Table 2).

**Table 2:** The magnitude of undiagnosed diabetes and hypertension in relation with different variables among patients with severe mental illness receiving antipsychoitic agents

Variables	Category	Total n (%)	Prediabetes	Diabetes	Prehypertension	Hypertension
			Yes = n (%)	Yes = n (%)	Yes = n (%)	Yes = n (%)
Sex	Male	138 (58.2)	32 (13.5)	11 (4.6)	32 (13.5)	3 (1.3)
	Female	99 (41.8)	27 (11.4)	4 (1.7)	23 (9.7)	8 (3.4)
Age, years	≤ 40	195 (82.3)	41 (17.3)	9 (3.8)	39 (16.5)	6 (2.5)
	> 40	42 (17.7)	18 (7.6)	6 (2.5)	16 (6.8)	5 (2.1)
BMI, Kg/m^2^	< 25	184 (77.6)	41 (17.3)	10 (4.2)	34 (14.3)	1 (0.4)
	≥ 25	53 (22.4)	18 (7.6)	5 (2.1)	21 (8.9)	10 (4.2)
WC	Normal	209 (88.2)	50 (21.1)	13 (5.5)	47 (19.8)	2 (0.8)
	Abnormal	28 (11.8)	9 (3.8)	2 (0.8)	8 (3.4)	9 (3.8)
Marital status	Unmarried	143 (60.3)	27 (11.4)	6 (2.5)	27 (28)	4 (1.7)
	Married	94 (39.7)	32 (13.5)	9 (3.8)	28 (11.8)	7 (3.0)
Physical exercise	Sedentary	203 (85.7)	53 (22.4)	13 (5.5)	49 (20.7)	9 (3.8)
	Moderate	34 (14.3)	6 (2.5)	2 (0.8)	6 (2.5)	2 (0.8)
Smoking	No	220 (92.8)	55 (23.2)	14 (5.9)	51 (21.5)	11 (4.6)
	Yes	17 (7.2)	4 (1.7)	1 (0.4)	4 (1.7)	0 (0.0)
Alcohol	No	223 (94.1)	58 (24.5)	14 (5.9)	55 (23.2)	11 (4.6)
	Yes	14 (5.9)	1 (0.4)	1 (0.4)	0 (0.0)	0 (0.0)
Khat chewing	No	216 (91.1)	55 (23.2)	14 (5.9)	53 (22.4)	9 (3.8)
	Yes	21 (8.9)	4 (1.7)	1 (0.4)	2 (0.8)	2 (0.8)
Disease duration	≤ 6 years	204 (86.1)	47 (19.8)	12 (5.1)	44 (18.6)	8 (3.4)
	> 6 years	33 (13.9)	12 (5.1)	3 (1.3)	11 (4.6)	3 (1.3)
HDL-c, mg/dl	Normal	140 (59.1)	34 (14.3)	10 (4.2)	38 (16.0)	2 (0.8)
	Abnormal	97 (40.9)	25 (10.5)	5 (2.1)	17 (7.2)	9 (3.8)
TG, mg/dl	< 150	173 (73)	37 (15.6)	11 (4.6)	39 (16.5)	5 (2.1)
	≥ 150	64 (27)	22 (9.3)	4 (1.7)	16 (6.8)	6 (2.5)
TC, mg/dl	< 200	178 (75.4)	38 (16.1)	9 (3.8)	41 (17.4)	6 (2.5)
	≥ 200	58 (24.6)	20 (8.5)	6 (2.5)	13 (5.5)	% (2.1)

BMI, body mass index; FBS, fasting blood sugar; TC, total cholesterol; TGs, triglycerides; HDL-c, HDL-cholesterol; WC, waist circumference; abnormal HDL-c is < 40 mg/dl in males and < 50 mg/dl in females

Regarding occupation, 4.2%, 5.1%, 8.4%, and 4.2% of prediabetes was observed among employed patients, housewife, jobless participants, and students, respectively. While the proportion of type-2 diabetes was, 2.5% among the employed group and 1.3% was seen in jobless participants. Pre-hypertension was 5.9% in the employed group, 5.1% in jobless participants, and 3.8% in students. Besides, the prevalence of HTN was 2.5% in jobless patients with SMI.

The prevalence of prediabetes, type-2 diabetes, prehypertension, and hypertension was higher among patients with schizophrenic disorder when compared to those with other mental disorders: the rates were 14.3%, 3.4%, 13.5%, and 2.5%, respectively (Table 3).

**Table 3:** The magnitude of undiagnosed diabetes and hypertension in relation to types of mental illness

Variables	Total	Prediabetes	Diabetes	Prehypertension	Hypertension
	n (%)	Yes = n (%)	Yes = n (%)	Yes = n (%)	Yes = n (%)
Schizophrenia disorder	112 (47.3)	34 (14.3)	8 (3.4)	32 (13.5)	6 (2.5)
Schizophrenic form disorders	9 (3.8)	6 (1.3)	0 (0.0)	1 (0.40	0 (0.0)
Schizoaffective disorder	1 (0.4)	0 (0.0)	0 (0.0)	0 (0.0)	0 (0.0)
Major depressive disorder with psychotic feature	86 (36.3)	26 (11)	5 (2.1)	20 (8.4)	5 (2.1)
Bipolar (I, II, cyclothymic) disorder	21 (8.9)	1 (0.4)	1 (0.4)	1 (0.4)	0 (0.0)
Delusional disorder	8 (3.4)	2 (0.8)	1 (0.4)	1 (0.4)	0 (0.0)

### The magnitude and correlations of undiagnosed type-2 diabetes and hypertension in relation to different variables

**Fig. 1:**
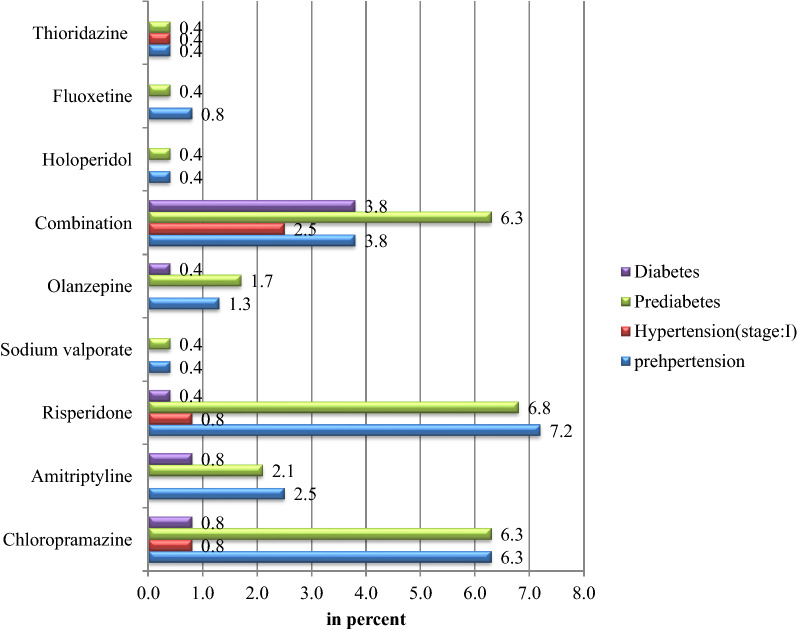
The magnitude of undiagnosed diabetes and undiagnosed hypertension in relation to antipsychotic treatments among psychiatric patients

The proportion of prediabetes among patients using chlorpromazine was 6.3%, risperidone (6.8%), and combination treatment (6.3%), while the type-2 diabetes rate was higher among patients using combined treatments (3.8%). Similarly, prehypertension rate among patients receiving chlorpromazine, risperidone and combined treatment was 6.3%, 7.2%, and 3.8%, respectively. While the rate of hypertension was higher among patients receiving combined treatment (2.5%) when compared to patients who were receiving other treatment groups (Fig. 1).

Table 4 shows the Pearson correlation of SBP, DBP and FBS with independent variables. SBP was significantly correlated with age (r = 0.25, p < 0.0001), duration since the diagnosis of disease (r = 0.15, p = 0.017), TG/HDL-c ratio (r = 0.147, p = 0.023), TC (r = 0.137, p = 0.035) and WC (r = 0.54, p < 0.0001). Besides, TGs and BMI also were significantly correlated with SBP. Age, TG/HDL-c, TC, WC, TGs, BMI, and non-HDL-c were significantly correlated with DBP. While FBS correlated with age, duration of diseases, TG/HDL-c, TC, HDL-c, TC/HDL-c, non-HDL-c, and LDL-c (Table 4).

**Table 4:** Pearson’s correlation of fasting glucose and blood pressure with independent variables

Variables	Mean (± SD)	Significance	SBP	DBP	FBS
Age	31.7 (10.9)	Correlation coefficient (r)	0.253	0.223	0.195
		P-value	< 0.0001	0.001	0.003
Duration since the diagnosis of disease	3.8 (2.7)	Correlation coefficient (r)	0.155	0.069	0.132
		P-value	0.017	0.291	0.042
TG/HDL-c ratio	2.9 (2.0)	Correlation coefficient (r)	0.147	0.118	0.161
		P-value	0.023	0.071	0.013
TC	168.2 (47.2)	Correlation coefficient (r)	0.137	0.151	0.257
		P-value	0.035	0.020	< 0.0001
WC	81.7 (10.5)	Correlation coefficient (r)	0.541	0.437	0.060
		P-value	< 0.0001	< 0.0001	0.359
HDL-c	50.3 (17.7)	Correlation coefficient (r)	0.026	0.054	0.033
		P-value	0.686	0.405	0.618
TGs	123 (58.6)	Correlation coefficient (r)	0.201	0.190	0.110
		P-value	0.002	0.003	0.091
BMI	22.9 (4.7)	Correlation coefficient (r)	0.447	0.396	0.040
		P-value	< 0.0001	< 0.0001	0.539
TC/HDL-c ratio	3.8 (1.8)	Correlation coefficient (r)	0.084	0.068	0.241
		P-value	0.95	0.30	< 0.0001
Non-HDL cholesterol	117 (47.2)	Correlation coefficient (r)	0.127	0.131	0.269
		P-value	0.051	0.04	< 0.0001
LDL-c	93.3 (44.3)	Correlation coefficient (r)	0.082	0.09	0.259^*^
		P-value	0.21	0.17	< 0.0001

BMI, body mass index; FBS, fasting blood sugar; TC, total cholesterol; TGs, triglycerides; HDL-c, HDL-cholesterol; WC, waist circumference; LDL-c, LDL-cholesterol

In linear regression analysis, all independent variables were significantly associated with FBS (p-value < 0.01). Out of lipid profiles, LDL-c and non-HDL-c were not deliberated as significant predictors. In multiple linear regression analysis: age, physical exercise, TG/HDL-c, HDL-c, and TGs were positively and significantly correlated with FBS, p-value < 0.05, while TG was negatively correlated with FBS (Table 5).

**Table 5:** Simple linear regression and multiple linear regression analysis with fasting blood sugar as a dependent variable

	Simple linear regression analysis for fasting blood sugar					Multiple linear regression analysis for fasting blood sugar				
Predictors	B	SE	β	t	p-value	B	SE	β	t	p-value
Sex of the participant	95.2	7.999	0.612	11.9	< 0.0001	− 6.533	3.617	− 0.042	− 1.806	0.072
Age of participant	2.78	0.073	0.928	38.29	< 0.0001	0.424	0.165	0.141	2.576	0.011
Alcoholism	89.14	26.27	0.21	3.93	0.001	− 1.874	7.867	− 0.005	− 0.238	0.812
Smoking	94.23	23.63	0.25	3.987	< 0.0001	− 8.614	7.221	− 0.023	− 1.193	0.234
Physical exercise	97.877	3.057	0.902	32.02	< 0.0001	10.773	4.812	0.099	2.239	0.026
Schizophrenia	100.15	6.929	0.685	14.45	< 0.0001	5.073	3.611	0.035	1.405	0.161
Drugs combination	98.89	9.15	0.575	10.8	< 0.0001	7.114	3.644	0.041	1.952	0.052
TC/HDL-c ratio	21.519	0.691	0.897	31.15	< 0.0001	1.148	3.252	0.048	0.353	0.724
TG/HDL-c ratio	23.35	1.079	0.815	21.63	< 0.0001	9.739	3.748	0.340	2.598	0.010
Waist circumference	1.169	0.023	0.958	51.58	< 0.0001	0.141	0.174	0.116	0.813	0.417
Body mass index	4.072	0.090	0.947	45.27	< 0.0001	0.066	0.454	0.015	0.146	0.884
LDL-cholesterol	0.877	0.028	0.901	31.86	< 0.0001	–	–	–	–	–
Disease duration	17.415	0.827	0.808	21.05	< 0.0001	0.837	0.642	0.039	1.305	0.193
HDL-cholesterol	1.707	0.052	0.907	33.0	< 0.0001	0.645	0.167	0.343	3.868	< 0.0001
Triglycerides	0.651	0.022	0.883	28.97	< 0.0001	− 0.237	0.098	− 0.322	− 2.413	0.017
Total cholesterol	0.544	0.012	0.947	45.21	< 0.0001	0.122	0.087	0.212	1.409	0.160
Non-HDL cholesterol	0.729	0.02	0.922	36.5	< 0.0001	–	–	–	–	–

LDL-cholesterol and Non HDL-cholesterol were excluded variables multiple linear analysis; model adjusted for Sex, alcoholism, smoking exercise, schizophrenia and drug combination; B, Unstandardized coefficients; β, Standardized coefficients; SE, standard error; [FBS, Multiple R: 0.97; R^2^: 0.941; Adjusted R^2^: 0.0.937; F: 234.5; degree of freedom: 15; *p* < 0.0001]

In addition, SBP and DBP were positively correlated with physical exercise, TC/HDL-c, WC, BMI, and HDL-c, p-value < 0.05. Whereas sex was negatively correlated with SBP and DBP (Table 6).

**Table 6:** Multiple linear regression analysis with blood pressure as a dependent variable

Predictors	Multiple linear regression analysis for SBP					Multiple linear regression analysis for DBP				
	B	SE	β	t	p-value	B	SE	β	t	p-value
Sex of the participant	− 3.646	1.487	− 0.020	− 2.452	0.015	− 2.963	1.087	− 0.024	− 2.725	0.007
Age of participant	0.131	0.068	0.037	1.940	0.054	0.094	0.049	0.040	1.900	0.059
Alcoholism	1.931	3.235	0.004	0.597	0.551	2.366	2.365	0.007	1.000	0.318
Smoking	− 0.489	2.969	− 0.001	− 0.165	0.869	− 0.750	2.171	− 0.003	− 0.346	0.730
Physical exercise	4.967	1.978	0.038	2.511	0.013	3.369	1.446	0.040	2.329	0.021
Schizophrenia	1.506	1.485	0.009	1.014	0.312	0.841	1.085	0.007	0.775	0.439
Drugs combination	2.027	1.498	0.010	1.353	0.177	1.207	1.095	0.009	1.102	0.272
TC/HDL-c ratio	3.225	1.337	0.112	2.412	0.017	2.444	0.977	0.130	2.501	0.013
TG/HDL-c ratio	1.471	1.541	0.043	0.954	0.341	0.672	1.127	0.030	0.596	0.551
Waist circumference	0.791	0.071	0.543	11.089	< 0.0001	0.477	0.052	0.499	9.151	< 0.0001
Body mass index	0.635	0.186	0.124	3.406	0.001	0.505	0.136	0.150	3.706	< 0.0001
Disease duration	0.141	0.264	0.005	0.535	0.593	− 0.095	0.193	− 0.006	− 0.493	0.622
HDL-cholesterol	0.567	0.069	0.252	8.261	< 0.0001	0.391	0.050	0.264	7.792	< .0001
Triglycerides	− 0.026	0.040	− 0.030	− 0.655	0.513	− 0.008	0.030	− 0.014	− 0.283	0.778
Total cholesterol	− 0.064	0.036	− 0.092	− 1.783	0.076	− 0.044	0.026	− 0.097	− 1.688	0.093

LDL-c and Non HDL-c were excluded variables from multiple linear analysis; model adjusted for Sex, alcoholism, smoking exercise, schizophrenia and drug combination. SBP, systolic blood pressure; B, Unstandardized coefficients; β, Standardized coefficients; SE, standard error; DBP, diastolic blood pressure; [SBP, Multiple R: 0.996; R^2^: 0.993; Adjusted R^2^: 0.993; F: 2093.1; degree of freedom: 15; *p* < 0.0001]; [DBP, Multiple R: 0.996; R^2^: 0.991; Adjusted R^2^: 0.991; F: 1685.8; degree of freedom: 15; *p* < 0.0001]

## Discussion

In this study, 6.3% (95% CI: 3.4–10.1) of patients with SMI receiving antipsychotic agents had undiagnosed type-2 diabetes. However, the finding was higher than the rate reported from community-based studies in South-Ethiopia [11], Amhara region [14], and an estimated national prevalence of DM in Ethiopia [28], in which the prevalence rate was 1.9%, 3.3%, and 4.36%, respectively. This variation might be attributed to the differences in the management of healthcare, access to health care, the effects of antipsychotic agents and lifestyles of the patients.

The proportion of type-2 diabetes in the present study was 6.3% and it was almost comparable with the reports of different studies conducted in psychiatric patients in national as well as international levels, like 6.1% in Southwest Ethiopia [29], 6.8% in the UK [30] and 6.5% in Holland [31]. However, it was lower than the reports of other studies that were conducted in psychiatric patients, for example 7.3% in Northwest Ethiopia [21], 8% in South Africa [10], 32% in California [32], 15.5% in refugee psychiatric patients [33], 16% in UK [9], 10.2% in USA [34] and 12.1% in Australia [35]. The study design, time duration since the diagnosis of SMI and treatment, generation of antipsychotic agents that have been received by patients and the number of study participants could be a possible reason for the variations.

The studies from Northwest Ethiopia [21] and China [36] reported that females with severe mental illness had a higher rate of undiagnosed diabetes when compared to males. This was not in line with the finding of the present study that revealed males had a higher rate of undiagnosed diabetes (4.6%) when compared to females (1.7%). About 17.4% of males had sedentary lifestyles when compared to females (4.2%) and this might be a possible reason for the variation of diabetes rate between sex in the depicted studies.

A review study revealed that the prevalence of type-2 diabetes among patients with schizophrenic disorders ranging from 10–15% [8]. While other studies reported 18.7% [36], 23.3% [37] and 23.9% with 2.4 times of more risks [38]. In this study, the proportione of type-2 diabetes among patients with schizophrenic disorder was 3.4%. The finding was almost comparable with the study conducted by Correll et al. [39], which indicated 3% of diabetes mellitus among schizophrenic patients.

In this study, 24.9% (95% CI: 19.4–30.4) of psychiatric patients had prediabetes/impaired fasting glucose and this in line with the study conducted in Southwest Ethiopia, which was 26.9% [28]. However, low rates were reported by different studies, like 4% [40] and 7% [41]. The differences might be attributed to the individuals’ lifestyle, the diagnostic variability and the scale/guideline that applied to define hyperglycemia (National cholesterol education adult treatment panel III vs. the American Diabetes Association).

Concerning antipsychotic treatment agents, we found 6.3%, 6.3%, 6.8% 3.8% of patients had impaired fasting glucose among those who were receiving combination treatment, chlorpromazine, and risperidone, respectively. In support, a review report revealed that chlorpromazine, risperidone and combined antipsychotic treatments alter glucose metabolism from intermediate to high levels [32].

In this study, age was positively and significantly correlated with fasting blood glucose. This finding was in line with the studies conducted by Akter et al. [42] and Zakewos et al. [11]. This might be an increasing body fat with aging, which may have an impact on the proper functioning of insulin and it may consequences insulin resistance. Further beta cells proliferation rate could be affected by the increase of the individuals’ age and it could results in more apoptosis than proliferation [43, 44].

In this study, the overall 4.6% (95% CI: 2.1–8.0) of psychiatric patients had HTN and the finding was lower than the prevalence reported from different studies that conducted in psychiatric patients, for example, 32% [10], 13.2% [8], and 42% [33]. Besides, in this study, 23.2% (95% CI: 17.3–29.5) of psychiatric patients had prehypertension and it was higher than the study conducted by Correll et al. which was 10% [8]. The variations in raised blood pressure and hypertension status might be attributed to differences in the type of antipsychotic drugs that utilized by individuals, duration since the diagnosis of mental problems and treatments, and patients' lifestyle. Moreover, we found that sex, TC/HDL-c, HDL-c, physical activity, WC, and BMI also were significantly associated with BP. Similarly, different studies reported the association of hypertension with BMI [11, 13, 31, 45], hypertension with WC [11], and hypertension with cholesterol [46]. The alterations in the depicted parameters and physical inactivity in psychiatric patients might be suggestive them to develop cardiovascular complications, despite controlling the severity of mental problem through antipsychotic treatments. In support, psychotic patients have a low life expectancy and a high chance of mortality rate when compared with the general population [2, 3].

### Limitations of the study

First, the study did not consider the general population as the control group, however, we tried to compare our findings with previously conducted studies of the general adult population [11, 14] regarding diabetes and this might evidence patients with SMI have a higher rate of hyperglycemia/diabetes when compared to the control group. Second, this signifying the evidence of hypertension or diabetes and its causal risk factors adequately is impossible due to the cross-sectional nature of the study. Third, we used FBS to diagnose hyperglycemia/diabetes and an oral glucose tolerance test was not done due to procedural difficulties as well as the psychotic nature of patients. Irrespective of the depicted limits, this study ultimately adds supportive information in the scarce data condition of Ethiopia.

## Conclusion

The increasing trend of hyperglycemia, diabetes, and raised blood pressure were observed among psychiatric patients. The magnitude of type-2 diabetes among psychiatry patients receiving antipsychotics was higher than the rate of previously conducted several studies of the general populations. Besides, hypertension and undiagnosed type-2 diabetes were higher among patients receiving combined antipsychotic agents when compared to those receiving single type of treatment agents. Age, TG/HDL-c, HDL-c, physical exercise and TG were significantly associated with FBS, while TC/HDL-c, HDL-c, physical exercise, BMI, WC, and sex were associated with blood pressure.

Therefore, the findings indicate a need to assess blood glucose and blood pressure at baseline before the commencement of any antipsychotic therapy and during therapeutic follow up to manage any increasing trends. The findings also recommend the application of well-controlled cohort studies for the assessment of long-term effects of antipsychotic treatment and the disease itself on blood pressure and glucose level. Moreover, this study suggests a need to assess cost–benefit ratio analysis in the decision to treat serious mental illness against the equivalent side effects of medication.

## Data Availability

The dataset of this article is not openly available but it can be accessible on reasonable requests from the corresponding author with authorization of Hawassa University's comprehensive specialized hospital clinical director office.

## Abbreviations

BMI: Body mass index
CI: Confidence interval
HDL-c: HDL-cholesterol
WC: Waist circumference
TG: Triglycerides
SMI: Severe mental illness
SPSS: Statistical package for social sciences
